# Magnesium: A Magic Bullet for Cardiovascular Disease in Chronic Kidney Disease?

**DOI:** 10.3390/nu11020455

**Published:** 2019-02-22

**Authors:** Nicoline H. J. Leenders, Marc G. Vervloet

**Affiliations:** 1 Department of Nephrology, Amsterdam Cardiovascular Sciences, Amsterdam UMC, Vrije Universiteit Amsterdam, De Boelelaan 1117, 1081 HV Amsterdam, The Netherlands; n.leenders@vumc.nl; 2 Department of Physiology, Radboud Institute for Molecular Life Sciences, Radboud University Medical Center, Geert Grooteplein 28, 6525 GA Nijmegen, The Netherlands

**Keywords:** chronic kidney disease, magnesium, cardiovascular disease

## Abstract

Magnesium is essential for many physiological functions in the human body. Its homeostasis involves dietary intake, absorption, uptake and release from bone, swifts between the intra- and extracellular compartment, and renal excretion. Renal excretion is mainly responsible for regulation of magnesium balance. In chronic kidney disease (CKD), for a long time the general policy has been limiting magnesium intake. However, this may not be appropriate for many patients. The reference ranges for magnesium are not necessarily optimal concentrations, and risks for insufficient magnesium intake exist in patients with CKD. In recent years, many observational studies have shown that higher (in the high range of “normal” or slightly above) magnesium concentrations are associated with better survival in CKD cohorts. This review gives an overview of epidemiological associations between magnesium and overall and cardiovascular survival in patients with CKD. In addition, potential mechanisms explaining the protective role of magnesium in clinical cardiovascular outcomes are described by reviewing evidence from in vitro studies, animal studies, and human intervention studies with non-clinical endpoints. This includes the role of magnesium in cardiac arrhythmia, heart failure, arterial calcification, and endothelial dysfunction. Possible future implications will be addressed, which will need prospective clinical trials with relevant clinical endpoints before these can be adopted in clinical practice.

## 1. Background

**Figure 1:**
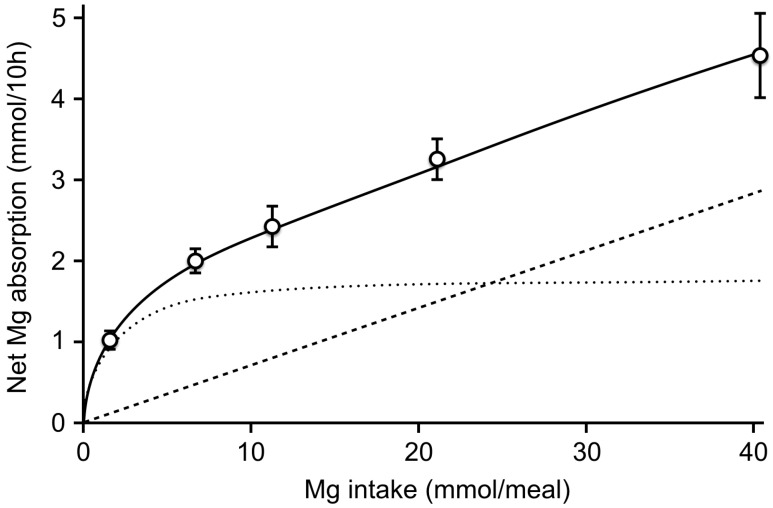
The relation between net magnesium absorption and dietary intake. The dashed lines represent the two types of absorption kinetics that explain net magnesium absorption. Reproduced with permission [7]. Copyright 1991, The American Society for Clinical Investigation.

Magnesium is the fourth most abundant cation and the second most abundant intracellular cation in the human body [1]. Magnesium is involved in many essential physiological functions. It is a co-factor for over 300 enzymatic reactions, many of which involve generation of adenosine triphosphate (ATP), it regulates transmembrane transport of other ions, including calcium and potassium, and stabilizes secondary structures of DNA and RNA [1]. Consequently, magnesium is essential for muscle contraction and relaxation, cardiac rhythm, vascular tone, neurological function, and cell proliferation. In the human body, the largest amount of magnesium is stored in bone (50–60%), muscle (20–30%), and other soft tissue (about 20%) [2]. Extracellular fluids contain about 1% of total body magnesium, including only 0.3% of total body magnesium in serum [1,2]. In serum, magnesium is present in three fractions: free ionized (60–70%), protein bound (20–30%), and complexed with anions (5–10%) [3]. The reference range for plasma magnesium can vary for different assays, but is typically 0.70–1.05 mmol/L. Homeostasis of magnesium involves dietary intake and absorption, uptake and release from bone, swifts between the intracellular and extracellular compartment, and renal excretion. Green leafy vegetables are rich in magnesium, as magnesium is in the center of the chlorine ring of chlorophyll molecules. Other foods with high magnesium content are nuts, seeds, unprocessed cereals, and cacao. Meat, starches, and milk have an intermediate magnesium content [4,5]. The recommended dietary allowance (RDA), defined as the average daily dietary intake level that is sufficient to meet the nutrient requirement of nearly all individuals, is 400–420 mg/day in adult men and 310–320 mg/day in adult woman depending on age group according to the U.S. Food and Nutrition Board [4]. Under normal dietary conditions, about 30% of ingested magnesium is absorbed, but fractional absorption increases if magnesium ingestion declines and can range from about 25–80% [6]. The pattern of the relation between dietary intake and enteral absorption is curvilinear, reflecting paracellular passive diffusion and transcellular transport via a saturable carrier (Figure 1) [7,8]. Most absorption of magnesium takes place in the small intestine [6]. Transcellular uptake is mediated by transient receptor potential cation channel subfamily M member 6 and 7 (TRPM6 and TRPM7), of which TRPM7 is ubiquitously expressed along the intestinal luminal membrane, whereas TRPM6 is predominantly expressed in the colon [8]. TRPM6 expression is upregulated by increased dietary magnesium intake, and this transporter is thought to be the main mediator of transcellular intestinal absorption [8]. Since enteral magnesium absorption is directly related to intake, renal excretion is mainly responsible for regulation of magnesium balance. At the glomerulus, non-protein bound magnesium (about 70–80% of total magnesium) is filtered. Different from many other ions and solutes, the proximal tubule is not the site where most reabsorption occurs. This is the thick ascending limb of the loop of Henle where about 60% of filtered magnesium is reabsorbed, while about 10% is reabsorbed in the distal convoluted tubule [9]. The kidneys can highly vary reabsorption to adapt to plasma magnesium levels.

In chronic kidney disease (CKD), an increase in fractional magnesium excretion compensates for declining glomerular filtration rate (GFR) in such a way that plasma magnesium concentrations can be maintained in the normal range for a long time with unchanged dietary intake [10]. In more advanced stages of CKD (from KDIGO stage 4 on), the increase of fractional excretion falls short and plasma magnesium levels usually rise, especially if GFR falls below 10 mL/min [10]. In end-stage kidney disease, magnesium excretion becomes dependent on dialytic clearance. Hemodialysis with a dialysate magnesium concentration of 0.75 mmol/L generally results in a stable plasma magnesium concentration ranging between 1.10 and 1.21 mmol/L [11,12]. A dialysate magnesium concentration of 0.50 mmol/L generally declines plasma magnesium concentration during dialysis with post-dialysis concentrations ranging between 0.67 and 1.09 mmol/L [11,13,14,15,16]. We recently showed that on hemodialysis with a dialysate magnesium concentration of 0.50 mmol/L, in the majority of patients an intra-dialytic decline of plasma magnesium concentration occurred, even if predialysis concentrations were as low as 0.75 mmol/L [16]. Only if pre-dialysis concentration was lower than that concentration, generally no additional decline was induced. For a long time, the emphasis in patients with CKD and those dependent on dialysis has been on the avoidance of magnesium loading. A single study that reported less adynamic bone disease after correcting hypermagnesemia in patients on hemodialysis treatment has also stimulated this approach [13]. However, this policy may not be appropriate for many patients, as will be outlined below.

The reference value for serum magnesium concentration is based on measurements of serum magnesium in representative healthy normal individuals from the NHANES I cohort in 1974 [17]. The reference range is, thus, based on prevalent concentrations, and dates back almost fifty years. In addition, these ranges did not take into account any outcome data and these are, therefore, not necessarily optimal concentrations, just as the prevalence of smokers in NHANES should not be the norm, since that norm also is not optimal. If magnesium homeostasis is disturbed, symptoms of hypo- or hypermagnesemia can develop. If magnesium concentrations are below the reference range, neuromuscular excitability, hypocalcemia, and hypokalemia can occur and if magnesium drops below 0.50 mmol/L more severe symptoms, including tetany, insults, and arrhythmia, have been described [18]. Reported symptoms of hypermagnesemia (when serum magnesium concentrations are above 2.0 mmol/L, substantially above the upper limit of the current reference range) include lethargy, drowsiness, flushing, nausea and vomiting, and diminished deep tendon reflexes [18]. If serum magnesium concentrations rise above 3.0 mmol/L, somnolence, loss of deep tendon reflexes, hypotension, and ECG changes can also occur, and in extreme hypermagnesemia (above 5.0 mmol/L) complete heart block, cardiac arrest, apnoea, paralysis, and coma have been reported [18]. However, symptoms of hypermagnesemia typically are not observed before plasma magnesium concentrations exceed 2.0 mmol/L [18]. In recent years, many observational studies have shown that higher (in the high range of “normal” or slightly above) magnesium concentrations are associated with better survival in the general population and also in CKD cohorts [19,20,21,22,23,24,25,26,27,28]. Therefore, magnesium has gained attention in the field of research, clinical medicine, and amongst patients. Attention to relatively low plasma magnesium concentrations in CKD patients may be needed. The diets of almost 50% of U.S. adults do not meet the estimated average requirement of magnesium [29]. The increasing predominance of ultra-processed foods can contribute to lower dietary magnesium intake, as dietary magnesium intake is inversely related to dietary share of ultra-processed foods [30]. In addition, the increased use of proton pump inhibitors in the general population exacerbates the tendency of lower gastrointestinal uptake since these drugs decrease enteral absorption of magnesium [31,32]. A final risk for insufficient magnesium intake exists in patients with CKD, because foods high in magnesium content frequently are also high in potassium content, and hence discouraged to use. In this review, we will describe recent observational studies that associate overall and cardiovascular survival with plasma magnesium concentration in patients with CKD, and provide an overview of possible underlying mechanisms that may drive this risk by reviewing available data from observational, in vitro, animal, and interventional studies.

## 2. Epidemiological Associations between Magnesium and Mortality in Patients with CKD

In recent years, multiple observational studies have shown that higher serum magnesium concentrations, notably even above the reference range, are associated with a lower all-cause mortality in patients treated with hemodialysis [19,20,21]. In a Japanese single center study in 515 patients on chronic hemodialysis without acute illness, significant infection, or malignancy, a mean serum pre-dialysis magnesium concentration of 1.14 mmol/L or above during a 4-month baseline period was statistically significantly associated with a lower all-cause mortality compared to those with a value below 1.14 mmol/L (*p* < 0.001) after a median follow-up of 51 (±17) months [19]. In addition, there was an inverse linear relation between serum magnesium and all-cause mortality that remained statistically significant after multivariable adjustment (adjusted hazard ratio (HR) 0.485 (0.241–0.975) per 0.411 mmol/L (1 mg/dL) increase of serum magnesium) [19]. Serum magnesium was not associated with cardiovascular mortality (HR 0.983 (0.313–3.086)) [19]. In a 48-month prospective cohort study in 206 patients on hemodiafiltration in a single center in Portugal, baseline pre-dialysis serum magnesium of 1.15 mmol/L or above was associated with a lower all-cause mortality in a multivariable adjusted model (adjusted HR 0.87 *p* = 0.01) [20]. This study did find a lower cardiovascular mortality in patients in the high serum magnesium category (adjusted HR 0.82 (0.72–0.95)) [20]. A European study in 515 patients on hemodialysis and hemodiafiltration also showed an inverse linear association between baseline serum magnesium and all-cause mortality that persisted beyond the upper reference range of serum magnesium in multivariable adjusted models after a mean follow-up of 37 (±21) months (maximally adjusted HR 0.88 (0.78–0.99) per 0.1 mmol/L increase of serum magnesium) [21]. In this study, there was a benefit in particular for cardiovascular mortality and sudden death (adjusted HR 0.73 (0.62–0.85) and 0.78 (0.66–0.92), respectively, per 0.1 mmol/L increase of serum magnesium) [21]. Another large Japanese cohort study among 142,555 patients treated with hemodialysis from a national renal data registry demonstrated a J-shaped relation between baseline serum magnesium and all-cause mortality, with the inflection point of lowest mortality at a serum magnesium concentration of 1.27 mmol/L [22]. The curve describing the relation between serum magnesium and cardiovascular mortality in this study had a similar shape and inflection point [22].

The association between serum magnesium concentration and cardiovascular mortality seems to be robust and remained after adjusting for multiple co-variables, including co-morbidity and surrogates of nutritional status (body mass index, albumin), in the studies described above. However, a study by Li et al. in 9359 incident patients on hemodialysis found a relationship between time-varying serum magnesium and all-cause mortality that lost significance in the maximally adjusted model after adding malnutrition-inflammation-cachexia syndrome-related factors, particularly serum albumin [33]. This may reflect the notion that hypomagnesemia may be causally related to malnutrition, which is very likely the case. In a subgroup analysis, a serum magnesium concentration below 0.82 mmol/L compared to a magnesium concentration above 0.82 mmol/L was associated with a higher mortality risk only in patients with a serum albumin concentration below 35 mg/dL (adjusted HR 1.17 (1.05–1.31)), reflecting that, especially in those at highest risk, magnesium appeared to be protective [33]. The absence of a protective effect of higher magnesium concentration in those with a serum albumin above 35 mg/dL is likely explained by the fact that these patients had a much lower a priori mortality risk (HR 0.4 compared to those with a lower albumin concentration) [33]. Two recent smaller studies did show a trend towards decreased all-cause mortality in patients with a higher serum magnesium concentration, but this trend was not statistically significant [34,35]. Besides the limited size of these study populations, in one study this may be because nonlinear associations were not taken into account and a reference category consisting of high magnesium concentrations was used, while the other study excluded patients with several co-morbidities, including severe cardiovascular disease. Importantly, despite excluding patients with these comorbidities in one study, both these studies did show a statistically significant inverse relation between serum magnesium concentrations and cardiovascular mortality.

A lower number of studies have been performed in patients treated with peritoneal dialysis, and data demonstrating the relation between serum magnesium and mortality are less robust in these patients compared to those on hemodialysis. Some studies have shown increased mortality in patients with hypomagnesemia compared to patients without hypomagnesemia or normal serum magnesium, but a benefit of high-normal or high magnesium concentrations compared to normal magnesium concentrations has not been demonstrated in these patients [24,25]. In a large cohort of 10,692 incident patients in the U.S. starting peritoneal dialysis, the relation between mean serum magnesium concentration in the first 91 days of treatment with all-cause mortality after a median follow-up of 13 months was studied [24]. In this study, serum magnesium below 0.74 mmol/L, compared to serum magnesium of 0.74 mmol/L or higher, was associated with all-cause mortality in a univariable analysis and in a case mix adjusted model (adjusted HR 1.23 (1.07–1.41)). In this study, events up to 60 days after switch to another dialysis modality were attributed to the peritoneal dialysis treatment, which may have influenced the results. Another study included 402 prevalent Chinese patients on peritoneal dialysis (median duration of peritoneal dialysis at enrolment 23 months (interquartile range (IQR): 12–38)) [25]. In this study, after a median follow-up of 49.9 months, only the lowest serum magnesium category at the time of inclusion compared to the highest magnesium category was significantly associated with all-cause mortality (HR 1.89 (1.04–3.42)) [25]. In a subgroup analysis stratified for gender, there only was a statistically significant relation between magnesium and all-cause mortality in female patients, indicating interaction between gender and magnesium [25], a finding not confirmed by other studies.

In patients with chronic kidney disease not treated with dialysis, an association between magnesium concentrations and mortality has also been demonstrated. A higher baseline serum magnesium concentration was associated with lower all-cause mortality in 1650 Belgian patients with mild CKD (mean estimated glomerular filtration rate (eGFR) 66.8 mL/min ± 32.6) after a median follow-up of 5.1 years (adjusted HR 0.930 (0.887–0.974) per 0.04 mmol/L increase of serum Mg). This association with all-cause mortality was confirmed in 306 patients with a virtually normal eGFR (89.3 mL/min/1.73 m^2^), from the Dallas Heart Study cohort, with oversampling of African-Americans and age between 30 and 65 years, after a median follow-up of 12.3 years (HR 1.25 (1.00–1.56) per 0.0822 mmol/L decrease of serum Mg) [26,27]. In this cohort, magnesium was not associated with the incidence of fatal and nonfatal cardiovascular events [27]. However, a study in 283 patients with CKD (eGFR 30–59, 15–29, and <15 (not on dialysis)) in, respectively, 101, 86, and 96 patients), that were included from the moment of first referral to a Turkish renal unit because of renal failure, did show a statistically significant inverse relation between serum magnesium and cardiovascular fatal and nonfatal events (adjusted HR 0.21 (0.10–0.46) per 0.411 mmol/L higher serum Mg) [36]. Another study did not find a statistically significant relation between serum magnesium concentration and all-cause mortality [37]. In this study, follow-up ended after occurrence of a nonfatal cardiovascular event and this approach may, thus, have missed subsequent events, including cardiovascular mortality and all-cause mortality, leading to bias in the analysis of all-cause mortality [37]. Collectively, the epidemiological studies demonstrate an association between a low serum magnesium concentration and both all-cause and cardiovascular mortality.

## 3. Potential Mechanisms Explaining the Role of Magnesium in Clinical Outcomes

### 3.1. Magnesium and Cardiac Arrhythmia

In patients with CKD, lower serum magnesium concentration has been associated with increased risk for sudden death [21]. This cause of death is generally considered to be the consequence of cardiac dysrhythmia. The relation between serum magnesium concentration and nonfatal cardiac arrhythmia in patients with CKD, however, has not been studied extensively, as it has been in subjects without CKD. In a prospective community-based cohort study, the ARIC-study, the risk for incident atrial fibrillation was higher in the lowest quintile of serum magnesium (<0.78 mmol/L) compared to the middle quintile (≥0.80–0.83) (HR 1.34 (1.16–1.54)) [38]. In the other quintiles of serum magnesium, the risk for atrial fibrillation did not differ significantly compared to the middle quintile [38]. In another prospective cohort study, the Framingham Heart Study, a population without previous cardiovascular disease, the lowest quartile of serum magnesium (≤0.73 mmol/L) was associated with an approximately 50% higher risk of incident atrial fibrillation compared to the highest quartile of serum Mg (≥0.82 mmol/L) (HR 1.52 (1.00–2.31)) [39]. In the second and third quartile, the incidence of atrial fibrillation was not significantly different compared to the highest quartile [39]. In another analysis of this cohort, with a mean serum magnesium concentration of 0.95 mmol/L (±0.08), a 0.08 mmol/L lower serum magnesium concentration was associated with a higher risk for complex or frequent premature ventricular complexes (PVCs) in a model adjusted for serum potassium and other risk factors for ventricular arrhythmia [40]. Complex or frequent PVCs predicted prognosis, including all-cause mortality in the general population [41]. Extrapolating this finding to the observation from the Framingham Heart Study suggests that magnesium deficiency may contribute to mortality. However, it is unknown what mechanisms, clinically operational in human cardiac tissue, may underlie the associations found between magnesium concentration and arrhythmia or sudden cardiac death. Hypomagnesemia is associated with electrographic disturbances, including tachyarrhythmia and prolonged duration of repolarization [18]. However, it is often difficult to ascribe specific electrocardiographic changes to magnesium disturbances because these often come together with imbalances of other electrolytes, including potassium [18].

**Figure 2:**
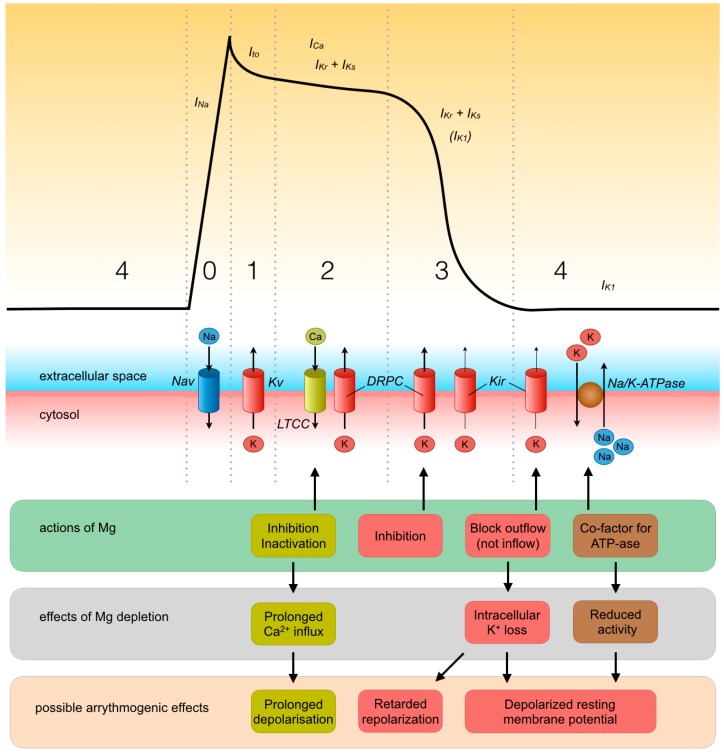
Cardiac excitation and potential effects of magnesium depletion. Processes during the different phases of the action potential are shown. I_Na_: inward sodium current; I_to_: transient outward potassium current; I_Ca_: inward calcium current; I_Kr_ + I_Ks_: outward potassium current through delayed rectifier potassium channels; I_K1_: potassium current through inwardly rectifying potassium channels; Nav: voltage-gated sodium channel; Kv: voltage-gated potassium channel; LTCC: L-type calcium channel; DRPC: delayed rectifier potassium channel; Kir: inwardly rectifying potassium channel; Na/K-ATPase: sodium/potassium-ATPase.

From in vitro studies, it is known that magnesium is extensively involved in the molecular mechanisms of cardiac excitation (Figure 2). First, magnesium has a role in establishing, and the maintenance of, the resting transmembrane potential. As magnesium is an essential co-factor for enzymatic reactions involving ATP, it is a critical co-factor for the Na/K-ATPase, which is involved in establishing the resting transmembrane potential. Indeed, hypomagnesemia decreases the activity of Na/K-ATPase in rat ventricle [42]. This decreased activity of Na/K-ATPase may decrease the intracellular potassium concentration, which brings the resting membrane potential more close to the threshold potential, promoting hyperexcitability. Normally, inwardly rectifying potassium channels (Kir) help to maintain the transmembrane potential and prevent excessive loss of potassium during repeated or prolonged action potentials as a result of their ability to carry inward potassium currents better than outward potassium currents [43]. Intracellular Mg is responsible for this inward rectifying function because internal Mg blocks the outward potassium currents but is extruded from the pore of the channel by inward potassium currents [44]. This function is lost if intracellular magnesium is depleted, enabling a larger outward potassium current and loss of intracellular potassium [44]. Loss of intracellular potassium caused by both a decreased activity of Na/K-ATPase and loss of potassium via inappropriately functioning Kir-potassium channels can result in depolarization of the membrane potential, leading to hyperexcitability of cardiomyocytes, which can increase the risk for development of arrhythmia.

Second, magnesium regulates the function of multiple other ion channels during the course of the action potential as well. Under physiological conditions, during the action potential of cardiomyocytes, an inward calcium current (I_Ca_) through L-type calcium channels helps to maintain depolarization during the plateau phase (phase 2) [45]. This occurs after the cardiomyocyte has been depolarized (phase 0) by an inward sodium current (I_Na_) through voltage-gated sodium channels (Nav), followed by a first small repolarization phase (phase 1) mediated by a transient outward potassium current (I_to_) through voltage-gated potassium channels (Kv) [45]. The inward calcium current is stopped at the end of the plateau phase when the L-type calcium channels close, because the influx of positively charged ions has increased the positive charge inside the cardiomyocyte (voltage-gated inactivation). Intracellular magnesium concentrations affect the function and inactivation of L-type calcium channels, as was shown in patch-clamp recordings of mice ventricular cardiomyocytes [46]. Increases in intracellular magnesium concentration reduce inward calcium currents and hasten voltage-gated inactivation of L-type calcium channels [46]. In conditions of magnesium depletion, the voltage-gated inactivation of L-type calcium channels occurs more slowly [46]. As a consequence, magnesium depletion may prolong the depolarization time and make the heart more vulnerable to arrhythmia. After the plateau phase of the action potential, repolarization (phase 3) is mediated by outward potassium currents (I_Kr_ and I_Ks_) through delayed rectifier potassium channels. Magnesium inhibits delayed rectifying potassium channels; thus, repolarization is retarded in hypermagnesemic conditions, as was shown in guinea pig cardiomyocytes [47]. Although these channels are not inhibited by low magnesium concentrations, the duration of depolarization may also be prolonged in hypomagnesemic conditions because magnesium depletion may reduce intracellular potassium concentrations (as described before) and thereby decrease the speed of potassium efflux during the depolarization phase. A prolonged duration of depolarization in hypomagnesemic (and hypermagnesemic) conditions may also increase the risk for development of arrhythmia.

In a study in hypomagnesemic Sprague Dawley rats, arrhythmia and sudden cardiac death occurred in rats on a magnesium-deficient diet with a mean serum magnesium concentration of 0.25 (±0.15) mmol/L, but not in rats on a normal magnesium diet with a mean serum magnesium concentration of 0.60 (±0.45) mmol/L [48]. However, in another study in rats, the intracellular magnesium concentration of ventricular cardiomyocytes was not changed in magnesium-deficient rats with serum magnesium of 0.29 (±0.03) mmol/L compared to normomagnesemic rats with a serum magnesium concentration of 0.86 (±0.07) mmol/L [49]. The combined results of these studies suggest that in the rats, a lower extracellular magnesium concentration dominates over the intracellular content in terms of inducing arrhythmia.

Intervention studies in CKD patients studying the effect of magnesium on arrhythmia are sparse. One prospective cross-over intervention study in 23 patients on hemodiafiltration showed that, by lowering the dialysate magnesium concentration from 0.50 mmol/L to 0.25 mmol/L, predialysis magnesium concentrations decreased from 0.99 (±0.16) to 0.81 (±0.08) mmol/L, and postdialysis from 0.87 (±0.11) to 0.56 (±0.06) mmol/L [50]. This acute decrease of magnesium by the low dialysate magnesium concentration was associated with a higher number of ventricular arrhythmias compared to the normal dialysate magnesium concentration, although statistical significance was not reported [50].

### 3.2. Magnesium and Heart Failure

Another mechanism involved in cardiovascular mortality in patients with CKD is cardiac remodeling and congestive heart failure. The effects of magnesium on these mechanisms also provide possible clues for a causal explanation of the association between serum magnesium and cardiovascular mortality in CKD patients. In patients with CKD, a lower serum magnesium is associated with a higher left ventricular mass index in univariable and multivariable cross-sectional analyses [20,51]. Prospective observational studies have been performed in non-CKD populations, relating lower serum magnesium concentration to a higher risk of incident heart failure and increase of left ventricular mass index [52,53,54]. In 14,709 patients from the ARIC cohort with no previous history of heart failure, there was a linear inverse association between serum magnesium concentration and incident heart failure after a median follow-up of 20.6 years (HR 1.71, (1.46–1.99) in the lowest (<0.7 mmol/L) of five magnesium categories versus the highest magnesium category (>0.9 mmol/L)) [52]. In a study of 3523 older men aged 60–79 years without prevalent heart failure or myocardial infarction, a higher serum magnesium was associated with a lower risk for incident heart failure after adjustment for conventional cardiovascular risk factors and incident myocardial infarction, and compared to the lowest quintile this was statistically significant in the highest quintile (HR 0.56 (0.36–0.86)) [53]. Another study, including 1202 inhabitants of Germany, showed that serum magnesium concentration was statistically significantly inversely associated with the increase of left ventricular mass index over 5 years of follow-up in a multivariable analysis [54].

Besides its role in mechanisms involved in arrhythmia (as described in Section 3.1) and coronary artery disease and calcification (as described in Section 3.3), which can be involved in the aetiology of heart failure, magnesium is also directly involved in mechanisms of cardiac remodeling, excitation–contraction coupling, and contraction and relaxation of cardiomyocytes. Pathological cardiac remodeling involves structural, functional, and molecular changes of the heart in response to pathogenetic stimuli. Fibroblasts present in heart tissue are the main producers and regulators of the extracellular matrix components of the myocardium. In primary rat fibroblast cultures, an increase of extracellular magnesium concentration inhibited expression of matrix metalloproteinase-2 (MMP-2), which is capable of cleaving components of the extracellular matrix, a process that contributes to cardiac remodeling [55].

**Figure 3:**
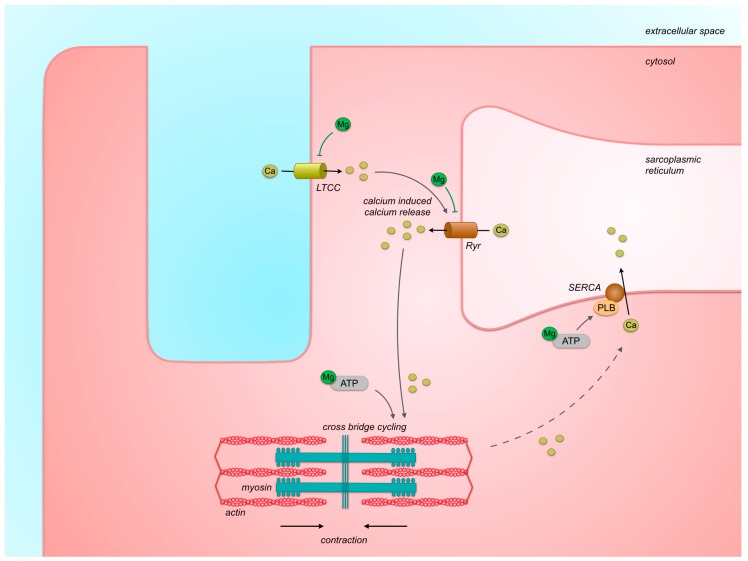
Cardiac excitation–contraction coupling. After initial depolarization, calcium influx via L-type calcium channels triggers calcium release from the sarcoplasmic reticulum via Ryanodine receptors. The amplified cytosolic calcium concentration triggers cross-bridge cycling of actin and myosin filaments leading to muscle contraction. Re-uptake of calcium into the sarcoplasmic reticulum via the phosphorylated-phospholamban-activated SERCA-pump leads to muscle relaxation. Actions of magnesium are shown. Arrows indicate stimulating effects, and right-angled line endings indicate inhibiting effects. LTCC: L-type calcium channel, Ryr: ryanodine receptor, PLB: phospholamban, SERCA: sarco/endoplasmic reticulum calcium ATP-ase.

Excitation–contraction coupling is the process in which electrical current (depolarization) induces mechanical muscle contraction [43] (Figure 3). Following depolarization of the cell membrane, L-type calcium channels mediate inflow of calcium into the cytoplasm during the plateau phase of the action potential. As mentioned before, magnesium stimulates voltage-dependent inactivation of these channels at the end the plateau phase, after which the influx of calcium is stopped [46]. The increase of cytoplasmic calcium concentration triggers release of calcium stored within the sarcoplasmic reticulum via the sarcoplasmic Ryanodine receptors (calcium-induced calcium release). In resting muscle in the absence of high calcium concentrations, magnesium inhibits activation of the Ryanodine receptor, as was demonstrated in single-channel studies of Ryanodine receptors from sheep heart using artificial lipid bilayers [56]. The amplified cytosolic calcium concentration resulting from activation of the ryanodine receptor induces heart muscle contraction because calcium ions bind to muscle filaments and enable cross-bridge cycling of actin and myosin muscle filaments. ATP and magnesium are needed to provide the energy for this cross-bridge cycling. During diastole, calcium flows back into the sarcoplasmic reticulum via sarco/endoplasmic reticulum calcium ATP-ase (SERCA), an ATP-driven calcium pump, which induces cytoplasmic calcium decay, leading to muscle relaxation. Based on these mechanisms, magnesium depletion may result in a persistent release of calcium from the sarcoplasmic reticulum via Ryanodine receptors during diastole, thereby inappropriately stimulating contraction during diastole, which can lead to diastolic heart failure (the “Leaky Ryanodine receptor” hypothesis) [57]. As magnesium is a co-factor for enzymatic reactions involving ATP, magnesium depletion may also disturb the reuptake of calcium into the sarcoplasmic reticulum via the ATP-driven SERCA pump. This may also result in insufficient relaxation during diastole and diastolic heart failure. In addition, this retarded cytoplasmic calcium decay may reduce calcium release in the subsequent cardiac cycle, resulting in reduced contractility and systolic heart failure. Also, sufficient magnesium concentrations are needed to enable the ATP-dependent contraction of muscle fibers. On the contrary, magnesium overload may also be harmful because this can reduce the influx of calcium via the L-type calcium channels that is responsible for excitation–contraction coupling [46].

Thus, magnesium is involved in many ways in the process of excitation–contraction coupling and relaxation of the cardiomyocyte and skeletal muscle, as shown in vitro. Suboptimal magnesium concentration may disturb these calcium fluxes and thereby contribute to heart failure. Based on the above-mentioned mechanisms, intracellular hypomagnesemia may disturb relaxation during diastole and in addition reduce contractility during systole. However, the cumulative effect of the above-described roles of magnesium in vivo is not known, especially the effects of specific magnesium concentrations on heart failure in patients with CKD. There are no intervention studies on the effect of magnesium supplementation on heart failure in chronic kidney disease.

### 3.3. Magnesium and Vascular Calcification

Vascular calcification is an important pathophysiological process in chronic kidney disease and a strong predictor of mortality [58]. Observational studies have shown that serum magnesium concentrations associate inversely with vascular calcification. In 1987, Meema et al. demonstrated that serum magnesium concentrations were lower in patients treated with peritoneal dialysis that had progressive peripheral artery calcifications on follow-up X-rays of hands, feet, and ankles, compared to patients with stable or improved calcifications (1.11 ± 0.21 versus 1.24 ± 0.21 mmol/L, *p* < 0.001) [59]. Thereafter, other studies have shown that lower serum magnesium is also associated with vascular calcification after multivariable analysis. In one study, lower serum magnesium was associated with an abdominal aortic calcification score on lateral lumbar spine radiographs in a cross-sectional analysis in patients treated with peritoneal dialysis [60]. In another study, lower serum magnesium was associated with 1-year progression of the aortic calcification index on abdominal computed tomography (CT) in a retrospective analysis in patients on hemodialysis or hemodiafiltration [61]. A third study showed that lower serum magnesium concentration was associated with a high vascular calcification score based on X-rays of pelvis and hands in a prospective study in patients on hemodiafiltration [20]. Serum magnesium concentration was also inversely associated with coronary artery density in a cross-sectional analysis in patients with CKD, although it was not associated with total coronary artery calcification score (Agatston score) or with progression of this score during follow-up in patients treated with hemodialysis [62,63]. Moreover, lower magnesium was also associated with higher carotid intima media thickness (IMT) in a cross-sectional study in patients with stage V CKD in type 2 diabetes mellitus patients with mild to moderate CKD (eGFR 15–80 mL/min), and in patients on hemodialysis [51,64,65]. One study, however, after a univariable analysis in patients treated with hemodialysis, found no statistically significant association of serum magnesium with IMT [66]. Besides calcification and IMT, lower serum magnesium was also associated with higher pulse wave velocity, an indicator of arterial stiffness, in patients with end-stage CKD, including those treated with hemodialysis [64,66].

**Figure 4:**
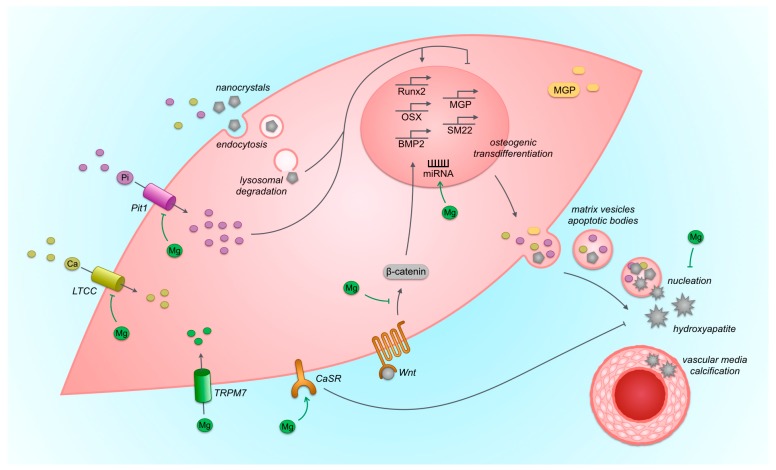
Vascular smooth muscle cell calcification. Actions of magnesium that may explain inhibiting effects of magnesium on vascular calcification are shown. Arrows indicate stimulating effects, and right-angled line endings indicate inhibiting effects. LTCC: L-type calcium channel, Pit1: phosphate transporter Pit1; TRPM7: transient receptor potential cation channel subfamily M member 7; CaSR: calcium sensing receptor; Wnt: Wnt protein; Runx2: runt-related transcription factor 2; OSX: osterix; BMP2: bone morphogenetic protein-2; MGP: matrix gla protein; SM22: transgelin (smooth muscle specific protein); miRNA: micro RNA.

Vascular calcification in chronic kidney disease is associated with mineral disturbances, including high phosphate and calcium concentrations [67], loss of mineralization inhibitors, including carboxylated matrix gla protein (carboxylated MGP) and fetuin A [68,69], damage to and apoptosis of vascular smooth muscle cells, and an active process of osteogenic transformation of vascular smooth muscle cells (VSMCs) [70]. In this milieu of mineral disturbances and loss of mineralization inhibitors, calcium-phosphate nanocrystals can be formed [71] (Figure 4). These crystals are taken up by VSMCs probably via endocytosis, followed by lysosomal degradation resulting in intracellular release of calcium and phosphate [72]. In addition, calcium can be taken up by the cell via calcium channels and phosphate is taken up via phosphate transporters (Pit1) [73]. Calcium-phosphate nanoparticles and phosphate induce expression of osteogenic transcription factors including runt-related transcription factor 2 (Runx2) and bone morphogenetic protein-2 (BMP2); genes associated with mineralization of the extracellular matrix including alkaline phosphatase (AF); and decrease expression of calcification inhibitory proteins including MGP [71,74]. In response to elevated extracellular concentrations and intracellular release of calcium and phosphate, decreased serum levels of calcification-inhibiting factors (including Fetuin A), and stimulated by osteogenic transformation, VSMCs secrete matrix vesicles containing calcium/phosphate nanocrystals and depleted from mineralization inhibitors (including MGP) [75,76]. The intracellular calcium burst triggers apoptosis and this is associated with release of apoptotic bodies containing calcium and phosphate [77]. Matrix vesicles and apoptotic bodies containing calcium-phosphate nanocrystals and decreased amounts of calcification inhibitors (including Fetuin A and MGP) provide a nidus for mineral nucleation and maturation [75]. This process includes transformation of primary calciprotein particles to secondary calciprotein particles [78]. The extracellular release of calcium and phosphate also results in a positive feedback loop with additional uptake of calcium-phosphate nanocrystals by VSMCs.

Dietary magnesium may counteract vascular calcification by inhibition of intestinal phosphate uptake as a result of phosphate binding, by systemic effects on both promoting and inhibiting factors of calcification, or by local effects at the vascular tissue level. Regarding the tissue level, magnesium counteracts calcification of vascular smooth muscle cells and aortic tissue in vitro and inhibits expression of osteogenic transcription factors, including RUNX2, Osterix, and BMP-2; and genes associated with matrix mineralization, including AF [64,79,80,81,82,83,84,85,86]. The local inhibiting effects of magnesium on vascular calcification may be mediated in multiple ways (Figure 4). First, magnesium passively interferes with the maturation of amorphous calcium/phosphate particles, thereby preventing the formation of stable hydroxyapatite [87]. Serum calcification propensity is a novel test that measures the time of conversion of primary to secondary calciprotein particles (T50). This time is prolonged if magnesium is added to the serum in vitro, indicating protection against the inherent tendency to calcify [88]. Second, magnesium inhibits influx of calcium via L-type calcium channels in VSMCs as outlined above, and this affects vascular tone [89]. It is plausible that inhibition of calcium influx channels limits the intracellular rise of calcium concentration that is associated with vascular calcification, which may inhibit possible stimulating effects of intracellular calcium on the calcification process. Indeed, the phenylalkylamine verapamil, an L-type calcium channel antagonist, inhibited calcification of bovine vascular smooth muscle cells, although this effect was not shown for another type of antagonist of this channel (the dihydropyridines nifedipine and nimodipine) [90]. Third, magnesium acts on the calcium-sensing receptor (CaSR). Besides on the prototypical tissue of the parathyroid gland, the CaSR is expressed on vascular smooth muscle cells as well. This expression is decreased in calcifying conditions, and stimulation of the CaSR by calcimimetics inhibits vascular smooth muscle cell calcification in vitro [91,92]. Also, in clinical trials, calcimimetics appear to retard cardiovascular calcification [93]. Magnesium may exert similar effects. Magnesium has been shown to upregulate the CaSR in the parathyroid gland and decrease PTH secretion, and in hemodialysis patients, serum magnesium concentration was inversely associated with serum PTH concentrations, but the effects of magnesium on the CaSR of VSMCs have not been studied [94,95]. Fourth, magnesium may have direct intracellular effects that inhibit osteogenic transformation, but the mechanisms are unknown. Proposed mechanisms are inhibition of Wnt/beta-catenin signaling, which is a mediator of osteogenic transformation, and attenuation of dysregulation of micro-RNAs that regulate gene expression [82,96]. The existence of an intracellular effect of magnesium on vascular calcification is supported by studies that demonstrated that the protective effect of magnesium on calcification of vascular smooth muscle cells disappeared when the magnesium channel TRPM7 is blocked by its inhibitor 2-APB [81,83]. Finally, magnesium has been shown to reduce apoptosis of VSMCs in calcifying conditions and magnesium stimulates proliferation of VSMCs [80,97]. However, it is not known whether, in calcifying conditions, the reduced apoptosis is the result of a direct effect of magnesium on apoptosis or just a phenomenon reflecting reduced calcification when the calcification itself was the primary inducer of apoptosis.

Besides effects at the tissue level, magnesium may also exert inhibitory effects on calcification at the intestinal level by chelating phosphate, or via systemic effects. These effects should be considered in interpretation of in vivo studies, where it can be difficult to determine to which extent each level contributes to the calcification inhibiting effect. In a rat model of adenine-induced CKD with calcitriol-induced vascular calcification, increased dietary magnesium content decreased development of aortic, iliacal, and carotid artery calcification [98]. Equally, in a 5/6-nephrectomy rat model of CKD combined with a high-phosphate diet and calcitriol to induce calcification, an increased dietary magnesium content (added as magnesiumcarbonate) decreased development of arterial calcification [99]. In addition, this study showed that the increased dietary magnesium content reduced expression of the osteogenic transcription factors RUNX2 and BMP2 in aortic tissue, and also decreased serum phosphate concentration. Of note, the preventive effect of magnesium on vascular calcification was not only mediated by intestinal phosphate-binding, since a follow-up experiment demonstrated that vascular calcification was also reduced when magnesium was given intraperitoneally (as magnesiumsulphate) [99]. Magnesium-containing phosphate binders have also shown beneficial effects on vascular calcification in animals [100,101]. The effect of calcium acetate/magnesium carbonate (Osvaren^®^) compared to a vehicle only was studied in rats with adenine-diet-induced CKD that received a diet with a high phosphate content [101]. In the animals treated with the magnesium-containing phosphate binder, serum phosphate was reduced, serum magnesium slightly increased, and abdominal aorta calcium-content (in mg/g wet tissue) was reduced by approximately 75% [101].

Intervention studies in patients with CKD have shown effects of magnesium on several measures of vascular calcification. In a non-controlled pilot study, seven patients on hemodialysis were treated with magnesium/calciumcarbonate at a mean dose of 700 mg elemental Mg per day and 1200 mg elemental calcium per day (titrated to achieve a serum phosphorus of 1.8 mmol/L) [102]. In this study, there was no significant progression of coronary artery calcification scores (CAC-score) after 18 months treatment compared to baseline. In a randomized controlled trial (RCT) comparing two phosphate binders in 59 patients on hemodialysis, those allocated to magnesiumcarbonate/calciumacetate (Osvaren^®^) compared to the group that received calciumacetate, after 12 months of treatment, tended to develop less progression of calcification, but this difference was not statistically significant, which was ascribed to the limited sample size [103]. Two studies showed an effect of magnesium-based interventions on IMT [104,105]. In a controlled trial in patients on hemodialysis, participants were randomized to magnesiumoxide 440 mg three times weekly or a placebo. Carotid IMT was decreased after 6 months compared to baseline in those treated with magnesiumoxide, and worsened in those allocated to the placebo [104]. In another RCT in patients on hemodialysis, comparing magnesiumcitrate/calciumcitrate with calciumacetate as phosphate binders for 2 months, bilateral carotid IMT decreased in patients that received the magnesium-containing binder, and increased in the comparator group [105]. In addition, an improvement of calcification propensity score (T50) by magnesium-based interventions was demonstrated in two randomized clinical trials [106,107]. After an increment of dialysate magnesium concentration from 0.50 mmol/L to 1.00 mmol/L during 28 days, the mean calcification propensity score of week 21 and 28 improved in the intervention group compared to baseline and compared to the control group that continued a dialysate magnesium concentration of 0.50 mmol/L [106]. Treatment with magnesiumhydroxide 360 mg two times daily during 8 weeks in patients with CKD stage III and IV improved the calcification propensity score after 8 weeks compared to baseline, and this change over time was statistically significantly better compared to the control group that received a placebo [107]. Since previous observational data have demonstrated that the calcification propensity score is a predictor of mortality in patients with CKD, it is tempting to assume that magnesium supplementation ultimately may improve clinical outcomes, but this obviously needs prospective clinical trials [108].

### 3.4. Magnesium, Hypertension, and Endothelial Dysfunction

Magnesium also affects blood pressure and endothelial function. Higher serum magnesium concentration is associated with improved endothelial dysfunction in patients with CKD, demonstrated by increased flow-mediated dilation of the brachial artery [36,109]. In patients without CKD, serum magnesium is inversely associated with incident hypertension [110].

Mechanisms from in vitro studies that can explain the association between magnesium and blood pressure include effects of magnesium on contractility of vascular smooth muscle cells in the arterial wall, and effects of magnesium on endothelial function, thereby influencing vasodilation and vasoconstriction. In vascular smooth muscle cells, magnesium inhibits the influx of calcium via L-type calcium channels, which reduces vascular tone [89]. At the endothelial level, acetylcholine-induced vasodilatation (via release of endothelium-derived vasorelaxant factors, including nitric oxide) is magnesium-dependent and enhanced by increased magnesium concentrations in vitro [111,112].

In animal studies, a magnesium-deficient diet in Wistar rats induced an increase of blood pressure compared to rats on a normal magnesium diet [113]. In rats with hypertension induced by administration of a mineralocorticoid in combination with salt loading (DOCA-salt hypertensive rats), dietary magnesium supplementation decreased blood pressure [114]. These findings, therefore, are in line with the above-described mechanisms on both endothelial cells and vascular smooth muscle cells.

A small randomized controlled trial in patients on a three-times weekly hemodialysis schedule that compared magnesiumoxide 440 mg three times per week in 29 patients with a placebo in 25 patients was unable to demonstrate any effect on flow-mediated dilation (FMD) after 6 months [104]. However, in these patients with a long history of CKD, and having reached end-stage renal disease, one can question the reversibility of this endothelial dysfunction by any means. Interventional studies in participants without CKD have shown variable results of magnesium supplementation on FMD and blood pressure. Recently, meta-analyses of these studies have been performed. The first included six randomized controlled trials in 262 patients (including the study by Mortazavi et al. mentioned above) and concluded that magnesium supplementation at a dose ranging from 107 to 730 mg/day for a median of 3 months statistically significantly increased FMD (mean difference 2.97% (0.23–5.70)) [115]. Another meta-analysis of 34 randomized controlled trials in 2028 patients with normotension (18 trials) or hypertension (16 trials) and without CKD showed that magnesium supplementation at a median dose of 368 mg/day for a median duration of 3 months statistically significantly reduced systolic and diastolic blood pressure (mean decline 2.00 mmHg (0.43–3.58) and 1.78 mmHg (0.73–2.82), respectively) [116]. Remarkably, besides the blood-pressure-lowering effects of magnesium supplementation in hypertensive patients, an increase in dialysate magnesium concentration in patients on hemodialysis prevented the incidence and severity of intra-dialytic blood pressure decline [11,117]. In a cross-over study, 14 patients on chronic hemodialysis were treated in random order with variable dialysate magnesium concentration (low magnesium 0.25, intermediate 0.50, and high 0.75 mmol/L) for 4 weeks for each dialysate, separated by an interval of 2 weeks, during which the intermediate dialysate concentration was used [11]. In this study, there was a statistically significant decline of intra-dialytic systolic blood pressure during treatment with the low and intermediate magnesium dialysate concentration compared to the higher concentration, and an intra-dialytic decline of diastolic blood pressure in the low compared to the intermediate and high dialysate magnesium concentration [11]. A retrospective study in 45 patients on hemodialysis, in which dialysate magnesium concentration was increased from 0.50 mmol/L to 1.00 mmol/L, showed a statistically significant decrease of the incidence of intra-dialytic hypotension (defined as a drop in systolic blood pressure of more than 20 mmHg and concurrent requirement of volume expansion) in the 12 months after compared to the 12 months before the switch (from 1.59% of dialysis sessions ± 0.34 to 1.08% ± 0.27, *p* = 0.04) [117]. This decline of intra-dialytic hypotension by increased dialysate magnesium concentrations may be explained by beneficial effects of the higher dialysate magnesium concentration on cardiac output (as explained in previous paragraph) in the absence of a significant decline of peripheral vascular resistance induced by the change of dialysate magnesium concentration [11].

### 3.5. Magnesium and Diabetes, Inflammation, and Lipid Profile

Magnesium may also influence the cardiovascular system via metabolic and inflammatory effects. Magnesium intake is inversely associated with incident type 2 diabetes mellitus in the general population, as was shown in a large meta-analysis of prospective cohort studies [118]. Lower serum magnesium concentrations have been associated with development of pre-diabetes in participants with normal baseline glucose concentrations derived from a population-based cohort [119]. Randomized controlled trials showed that dietary magnesium supplementation reduces plasma glucose concentrations in patients with pre-diabetes and hypomagnesemia [120], and improves insulin sensitivity, fasting glucose concentrations, and HbA1c concentrations in patients with type 2 diabetes mellitus [121].

Besides patients with diabetes, a cross-sectional analysis in patients with CKD showed that serum magnesium concentration also is inversely associated with C-reactive protein (CRP) concentrations, as a reflection of low-grade inflammation [122]. Magnesium supplementation decreases CRP concentration, as was shown in a meta-analysis of randomized controlled trials in non-CKD populations [123].

An association between serum magnesium concentration and lipid profile has not been demonstrated consistently. A meta-analysis of randomized controlled trials in individuals with or without diabetes did not show a statistically significant effect of oral magnesium supplementation on lipid profile [124]. However, in subgroups with hypercholesterolemia and hypertriglyceridemia, there was a lowering effect on low-density lipoprotein cholesterol and triglyceride levels, respectively, in this meta-analysis [124]. The effect on triglyceride concentrations was confirmed in a randomized controlled trial that was published thereafter by the same research group, which demonstrated a beneficial effect of oral magnesium supplementation on triglyceride concentrations in individuals with the metabolic syndrome [125].

The mentioned metabolic and inflammatory effects may provide an additional link between lower magnesium concentrations and cardiovascular complications. An extensive overview of these effects is beyond the scope of this review.

## 4. Conclusions and Future Perspectives

Serum magnesium concentration consistently is inversely associated with all-cause and cardiovascular mortality in patients treated with hemodialysis or peritoneal dialysis and patients with CKD not treated with dialysis. This association persists even above the “normal” reference range of magnesium concentrations. This suggests that the reference concentrations for serum magnesium do not necessarily represent the optimal concentrations, but should be higher in CKD. Conditions frequently existing in patients with CKD, including dietary potassium restriction, use of processed foods, use of proton pump inhibitors, and low dialysate magnesium concentrations, may put patients with CKD at risk for absolute or relative magnesium deficiency. Therefore, attention is needed for patients with CKD with relatively low serum magnesium concentrations.

Possible mechanisms underlying the inverse association between magnesium and clinical cardiovascular outcome include protective effects of higher magnesium concentrations on development of arrhythmia, heart failure, arterial calcification, hypertension, and endothelial dysfunction. In vitro studies and animal studies have shown protective effects of magnesium supplementation on many of these processes. In addition, intervention studies with intermediate outcome parameters also suggest beneficial effects of magnesium supplementation on the cardiovascular system. Prospective clinical trials are needed to determine the effect of magnesium supplementation on clinically relevant outcome and to determine how to optimize plasma magnesium concentrations in individual patients with CKD before individualized target ranges for serum magnesium concentrations can be adopted in clinical practice. If indeed lower serum magnesium concentration plays a causal role in cardiovascular complications, magnesium supplementation in these patients may provide a promising, yet simple and cheap novel tool to improve cardiovascular outcome.
